# Bio fabricated palladium nano particles using phytochemicals from aqueous cranberry fruit extract for anti-bacterial, cytotoxic activities and photocatalytic degradation of anionic dyes

**DOI:** 10.1039/d4ra03177f

**Published:** 2024-08-01

**Authors:** Edal Queen J., Augustine Arul Prasad T., Scholastica Mary Vithiya B., P. Tamizhdurai, Ghadah Shukri Albakri, Mohammad Khalid, Maha Awjan Alreshidi, Krishna Kumar Yadav

**Affiliations:** a PG and Research Department of Chemistry, Dwaraka Doss Goverdhan Doss Vaishnav College (Affiliated to University of Madras, Chennai) Arumbakkam Chennai 600106 Tamilnadu India augustineap@gmail.com; b PG and Research Department of Chemistry, Auxilium College (Affiliated to Thiruvalluvar University, Vellore) Gandhi Nagar Vellore 632006 Tamilnadu India; c Department of Teaching and Learning, College of Education and Human Development, Princess Nourah Bint Abdulrahman University P. O. Box 84428 Riyadh 11671 Saudi Arabia; d Department of Pharmaceutics, College of Pharmacy, King Khalid University Asir-Abha 61421 Saudi Arabia; e Department of Chemistry, University of Ha'il Ha'il 81441 Saudi Arabia; f Faculty of Science and Technology, Madhyanchal Professional University Ratibad Bhopal 462044 India; g Environmental and Atmospheric Sciences Research Group, Scientific Research Center, Al-Ayen University Thi-Qar Nasiriyah 64001 Iraq

## Abstract

The low cost and ecological compatibility of green technology makes it superior to chemical approaches in the generation of metal nanoparticles. The current study shows the use of cranberry fruit extract in the environmentally friendly green production of palladium nanoparticles. It is well known that the fruit extract from cranberries has a rich phytochemical composition that makes it a useful bio reducing agent for the formation of PdNPs. Several spectroscopic techniques, including ultraviolet-visible spectroscopy (UV-vis), X-ray diffraction (XRD), scanning electron microscopy (SEM), transmission electron microscopy (TEM), and energy-dispersive X-ray spectroscopy (EDX), were used to characterize the palladium nanoparticles (PdNPs). The diffractogram of the XRD analysis shows significant reflections at 39.98° (111), 46.49° (200), and 67.95° (220), which indicate the face-centered cubic (FCC) structure of PdNPs and demonstrate the crystallinity of the produced nanoparticles from the green method. The SEM and TEM structural and morphological analyses reveal that the synthesized nanoparticles have a spherical shape with size ranging between 2 nm to 50 nm. In addition, the synthesized PdNPs demonstrated possible antibacterial activity on both Gram-positive and Gram-negative bacteria as well as a cytotoxic effect on the MCF-7 breast cancer cell line. The degradation of Indigo Carmine (IC) and Sunset Yellow (SY) dyes can be effectively catalyzed by biogenic PdNPs, according to the results.

## Abstract 2

Biosynthesis of palladium nanoparticles employing aqueous cranberry fruit extract and their potential application as biological and catalytic agents.

## Graphical abstract

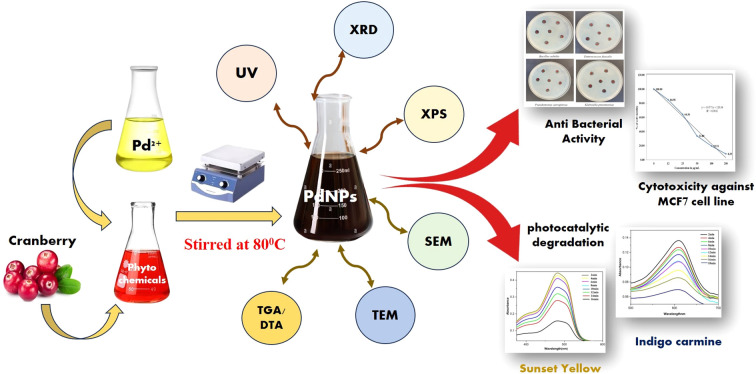

## 1. Introduction

With the advancement and growth of nanoscience, the 20th century has turned into a revolutionary one. The capacity to create metal nanoparticles from a variety of materials in a range of sizes and forms, as well as their successful integration into intricate structures, will determine how far this field advances.^1^ Compared to nanoparticles made using alternative physical and chemical synthesis techniques, biogenic nanomaterials provide additional advantages. Microorganisms, plants, and animals have all been used as reducing factors in the creation of metal nanoparticles. Because it is easy to do and environmentally friendly, plant-mediated biological synthesis of nanoparticles is becoming more and more popular.^2^ Because of the presence of many phytochemicals, plants have a strong potential to convert heavy metals from their higher oxidation state to zero oxidation state.

**Fig. 1:**
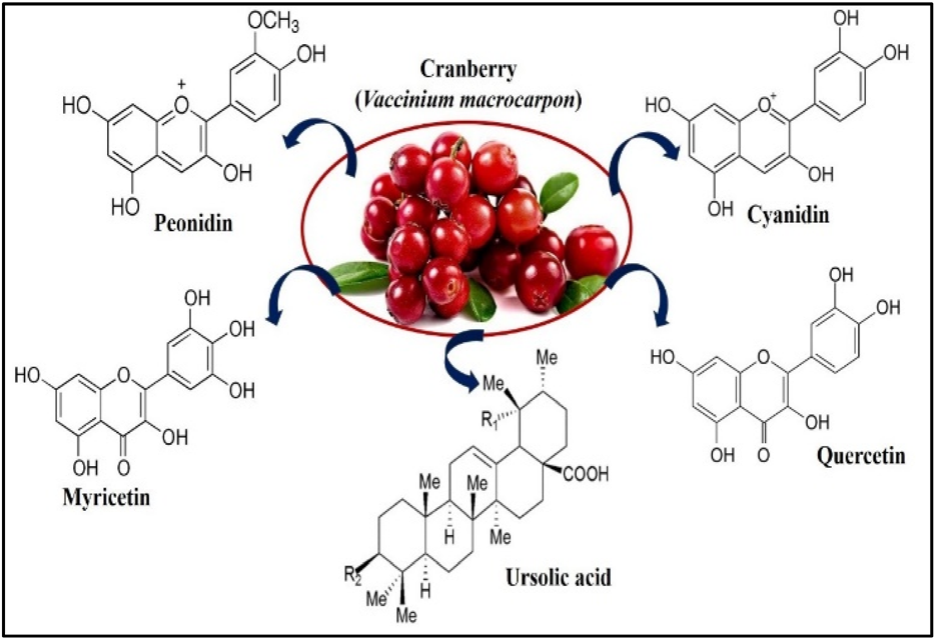
Cyanidin and peonidin are the major anthocyanin glycosides found in cranberry fruit. Quercetin glycosides are the major flavanols in cranberry fruit; myricetin glycosides are present in lesser quantities. The triterpenoid ursolic acid is also present in cranberry fruits.

Cranberry (*Vaccinium macrocarpon*) is a member of the Ericaceae family and contains a rich source of polyphenols, many other classes of phytochemicals and is abundant in bioactive compounds that have been associated with antibacterial, antiviral, anti-mutagenic, anti-carcinogenic, anti-tumorigenic, anti-angiogenic, anti-inflammatory, and antioxidant properties.^3^ They are frequently used to treat microbial infections in traditional folk medicine. The attractive bright red appearance and distinctive flavour of the cranberry are due to its unique flavonoids, including anthocyanins, proanthocyanidins, and flavanol glycosides. Some triterpenoids were also identified from cranberries, which have shown strong bioactivities against tumour cell proliferation.^4^ The major anthocyanins in cranberry (Fig. 1) are galactosides and arabinosides of cyanidin and peonidin. Quercetin is the major flavanol in cranberries and exists in several glycosidic forms (Fig. 1), primarily the 3-*O*-galactoside. Myricetin glycosides are also present in lesser quantity. Cranberry fruit contains a significant quantity of ursolic acid (UA) in its peel,^5^ in the aglycone form and as the cis and trans *p*-hydroxycinnamate esters, shown in Fig. 1.

The content of phenolic compounds in the cranberries is influenced by aspects such as cultivar, agriculture practices, geographical area, weather conditions, ripeness, harvesting time, and storage settings. Consuming cranberries can prevent tooth decay and gum disease, inhibit urinary tract infections, reduce inflammation in the body, maintain a healthy digestion system, and decrease cholesterol levels. This current work involves the biosynthesis of palladium nanoparticles (PdNPs) from the aqueous cranberry extract (ACE) for an effective and potential biological application as well as photocatalytic degradation of toxic organic dyes, since they are considered as most suitable owing to its resistance towards oxidation, long term stability and higher biocompatibility compared to other metal nanoparticles.^6^ Noble palladium nanoparticles have exceptional physicochemical characteristics, including high chemical and thermal durability, superior optical, plasmonic, and electronic properties, and high catalytic activity, when compared to other metallic nanostructures like Ag, Au, Cu, Mg and Pt.^7–10^ Palladium is also less expensive, easily accessible, and quickly produced in a variety of morphologies. Palladium nanoparticles are readily included or coated with various ligands or functional elements to create a range of sophisticated functional materials with desired characteristics. Pd NPs can be efficiently synthesized in a range of morphologies, including cubes, spheres, rods, flowers, octahedrons, and so on, because of their face-centered cubic (fcc) crystal structure. Pd is well-known among analytes for having a strong affinity for hydrogen (H_2_). In fact, it was the first metal to be used to reversibly introduce H_2_, and it has the capacity to absorb H_2_ at a rate of 900 times its equivalent volume.^11^

A green synthesis procedure has been developed to create palladium nanoparticles with regulated size and shape to fulfil numerous advanced applications, while chemical methods of producing palladium nanoparticles cause a harsh reaction and reduce the catalytic activity of palladium.^12^ The synthesis of Pd NPs *via* physical and chemical methods has various drawbacks. Energy-intensive instruments, such as those that need to sustain high temperatures or pressures, are needed for the physical approaches. Chemical processes like electrochemical deposition,^13^ sonochemical preparation,^14^ supercritical fluid nucleation,^15^ and wet chemical methods like the sol–gel method^16^ or the reduction by alcohols or other reductants, which may involve hazardous solvents and dangerous chemical reducing or stabilizing agents like sodium borohydride and hydrazine, can all result in the production of toxic pollutants and byproducts. Furthermore, the adsorption of chemical surfactants or hazardous compounds onto the surface of Pd NPs during their chemical manufacture might undermine their suitability for usage in pharmaceutical and biological applications.

**Fig. 2:**
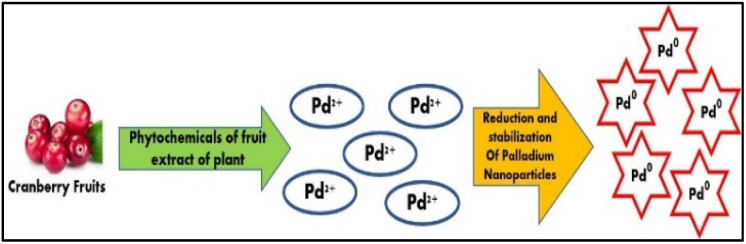
Graphical representation of the green synthesis of PdNPs using phytochemicals present in the aqueous cranberry fruit extract.

The biogenic production of PdNPs has the potential to offer easy, quick, affordable, and maybe ecologically favourable because the procedures rely on the utilization of biomolecules that are found naturally or metabolites from various organisms as stabilizing and reducing agents.^17^ Furthermore, it has been demonstrated that these biogenic processes provide extremely high levels of control on PdNPs characteristics including size and form.^18^ Using the aqueous cranberry fruit extract (ACE) in a one-step bio reduction approach is the least expensive way to create environmentally acceptable nanoparticles as shown in Fig. 2.

Additionally, the corresponding literature review's studies in (Table 1) shows that Cranberry (*Vaccinium macrocarpon*) has not yet been used in the synthesis of PdNPs, which piqued our intense curiosity in the current study. Thus, in this study, we herein report the synthesis and stabilization of palladium nanoparticles (PdNPs) using an aqueous extract of a cranberry fruit (ACE) for the first time and its characterization using different spectroscopic and optical techniques. The prepared PdNPs were characterized by UV-vis, TEM, EDS and FTIR analysis. Antibacterial and anti-cancerous potential of the PdNPs were also reported.

**Table 1:** Comparison of green synthesised PdNPs from various biological entity

Biological source	Reaction condition	Average size(nm)	Ref.
Annona squamosa peel	60 °C, 4 h	100	19
Solanum trilobatum leaf extract	Room temperature, 24 h	60–100	20
Banana peel	80 °C, 3 min	50	21
Catharanthus roseus leaf extract	60 °C, 2 h	38	22
Plectonema boryanum	25–100 °C, 28 days	<30	23
Cinnamom zeylanicum bark extract	30 °C, 72 h	15–20	24
Geobacter sulfurreducens	Room temperature, 3 h, anaerobic, electron donor and acceptor	5–10	25
Crane berry fruit extract	80 °C, 3 h	2–50	Present work

Catalytic reduction of organic dyes using metal nanoparticles is an intriguing topic, because of its great effectiveness, numerous industrial effluents released by the textile, cosmetic, leather, paper, food, and other chemical processing industries contain pigments and synthetic organic colours. Due to their extreme toxicity, industrial effluents tainted with dyes have a negative impact on both the environment and human health.^26^

Lastly, using NaBH_4_ as an electron donor at room temperature, the newly synthesized green PdNPs was used to degrade the dyes and proved to be more effective in degrading the more stable molecular structures of the anionic dyes sunset yellow (SY) and indigo carmine (IC).^27,28^ The current procedure that we have used, proved to be environmentally benign and economically viable photocatalyst that is ACE derived PdNPs for the degradation, the conditions for the effective photocatalytic degradation were optimized and the degradation was reached close to 100%.

The novelty of the current study lies in the fact that the ACE synthesized PdNPs with optimized synthetic route was effectively employed as a catalyst for dye degradation with significant optimization of parameters such as catalytic dosage, dye concentration and environmental pH. Furthermore, the promising results in cytotoxic effect on MCF-7 breast cancer cell line adds more value to the novelty of the present study.

## 2. Materials and methods

### 2.1 Chemicals

Palladium chloride (PdCl_2_) as the source of palladium ions, sodium borohydride (NaBH_4_), sunset yellow (SY) and indigo carmine (IC) were purchased from Sigma Aldrich and nutrient agar were purchased from HiMedia. All chemicals used in this study were of analytical grade. Double distilled water (DDW) from Merck Life Science (P) Ltd, were obtained and used in the preparation of the aqueous fruit extract and the green synthesis of palladium nanoparticles.

### 2.2 Preparation of aqueous cranberry fruit extract

Fresh cranberries (*Vaccinium macrocarpon* L.) were purchased from market, Chennai in Tamil Nadu, India. They were thoroughly cleaned to get rid of all dust and fungal spores using distilled water. To obtain the fruits clear aqueous extract, 10 g of fruit was crushed with 100 mL of double-distilled water and centrifuged. For later usage, it was kept in storage at 5 °C.

### 2.3 Preparation of bio genic palladium nanoparticles (PdNPs)

For preparation of PdNPs, 1 mL of aqueous cranberry extract was added to 10 mL of 1 mM aqueous solution of palladium(ii) chloride (PdCl_2_). At 80 °C, the extract was added dropwise while being constantly stirred in the dark. Three hours of continuous churning resulted in a color change of the nanoparticles from yellow to dark brown. The synthesis of PdNPs was indicated by a change in color in the aqueous fruit extract, which was further verified by UV-vis spectrophotometry operating in the 300–500 nm wavelength range. After that, the PdNPs were extracted by centrifuging it at 6000 rpm and drying it.

### 2.4 Antibacterial activity

The well diffusion method was used to assess the antibacterial activity of PdNPs synthesised from aqueous cranberry fruit extract. As test organisms, the Gram-positive bacterial strains of *Bacillus subtilis* (MTCC 443) and *Enterococcus faecalis* (MTCC 439) and Gram-negative bacterial strains of *Pseudomonas aeruginosa* (MTCC 96) and *Klebsiella pneumoniae* (MTCC 109) were employed. Strains that had developed over night were swabbed on the sterile Mueller Hinton agar Petri plate surface.^29^ After allowing the plates to set, 100 μL of the aforementioned bacteria that had grown for 18 hours (with an OD adjusted to 0.6) were placed onto a plate and used a sterile L-rod spreader to create a culture lawn. Following the bacteria' five-minute incubation period, a sterile cork-borer was used to create a 6 mm well on the agar. After being dissolved in sterile water, the test samples were added to wells in various combinations and concentrations. Azithromycin (30 μg mL^−1^) was used as the positive control and sterile water loaded well as the negative control. The plates were incubated for twenty-four hours at 37 °C in a bacteriological incubator. Using the antibiotic zone scale, the diameter of the zone of inhibition surrounding the well was measured to estimate the antibacterial activity.

#### 2.4.1. The mechanism underlying PdNPs' antibacterial action

**Fig. 3:**
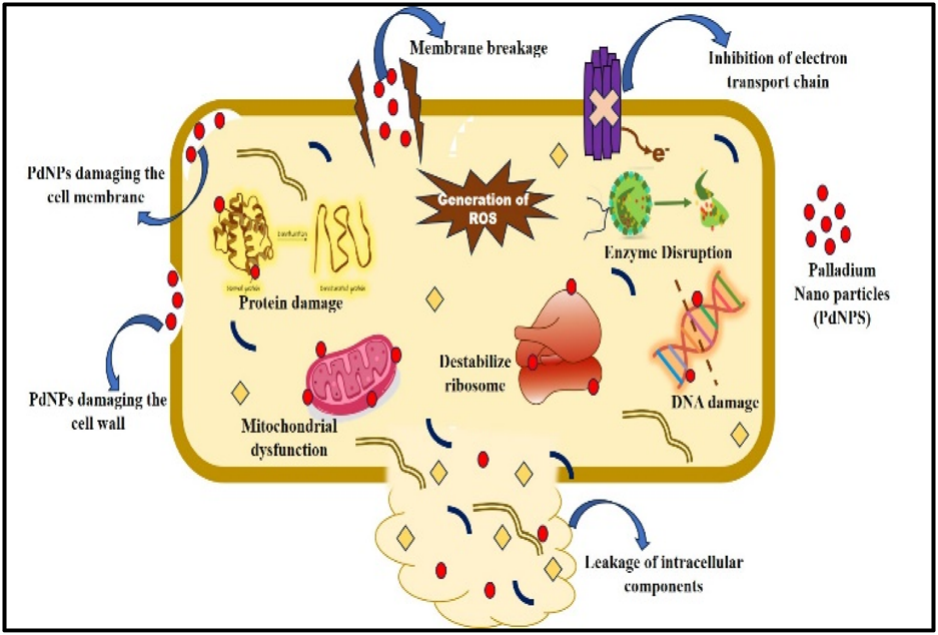
The plausible mechanism of PdNPs antibacterial activity.

Penetration of cells is the first stage in a microbial cell inhibition procedure of PdNPs. The primary method of NP penetration at the cell surface is adsorption or diffusion. Adsorption can occur when PdNPs adhere to the negatively charged functional groups of proteins, destroying the proteins and killing the cells.^29^ The generation of reactive oxygen species (ROS) and loss of cellular integrity are the underlying mechanisms that cause inhibition for bacteria. ROS produced by PdNPs interactions with bacteria cause oxidative stress, harming biomolecules, bacterial cell membranes, cellular structures, and envelopes.^30,31^ As shown in the Fig. 3 the bacterial cells are subjected to many modes of action by PdNPs, such as the destruction of their cell wall and membrane,^32^ inhibition of electron transport chain, leakage of intracellular components and the destruction of their constituent parts, degradation of ribosomes, suppression of DNA replication, malfunctioning of enzymes and prevention of biofilm development.^33^

### 2.5 Cytotoxicity on MCF-7 cell line

A slightly modified version of a previously published procedure was used to perform the MTT experiment.^34^ The stock cells of MCF-7 and A-375 cell lines were cultivated in 10% DMEM (Dulbecco's modified eagles medium, MP Biomedical; low glucose with glutamine), which was enhanced with foetal bovine serum (FBS). All cytotoxicity tests were conducted in 96 microtiters well plates, and the stock cultures were first grown in 25 cm^2^, then 75 cm^2^, and lastly 150 cm^2^ tissue culture flasks. 96 well-plates were filled with 2 × 10^4^ cells per well.

After being cleaned, cell lines in the exponential growth phase were again suspended in full culture media.^35^ A partial monolayer formed during the 24 hour incubation period after the cells were seeded at a density of 2 × 10^4^ cells per well in a 95-well microtiter plate. Next, different concentrations of the test chemicals were introduced to the cells. For a duration of 24 hours, the plates were incubated at 37 °C in a humidified incubator with 5% CO_2_ and 75% relative humidity. Using an inverted microscope, the morphological alterations of the drug-treated cells were analysed over a period of time. Cell viability was assessed using the 3-(4,5-dimethylthiazol-2-yl)-2,5-diphenyl tetrazolium bromide (MTT) test after a 24 hour period.

When mitochondrial succinate dehydrogenase and reductase of living cells break MTT, a quantifiable purple product called formazan is produced.^36^ The number of viable cells and the degree of cytotoxicity are directly and inversely correlated with the formazan production. The wells were supplemented with MTT after 48 hours of incubation and they were then left at room temperature for three hours. Using a pipette, the contents of each well were withdrawn. To dissolve the formazan crystals, 200 μL of SDS in DMSO was added. The absorbance was measured at 541 nm using a Lark LIPR-9608 microplate reader.

### 2.6 Catalytic activity of biogenic PdNPs

Experiments on the reductive degradation of anionic dyes, such as sunset yellow (SY) and indigo carmine (IC), were carried out using NaBH_4_ in the presence of biogenic PdNPs. The reduction of 1 mL of 0.01 mM anionic dyes SY and IC in aqueous solutions were examined for catalytic activity in the presence of 10 mM NaBH_4_. NaBH_4_ and biogenic PdNPs were added to the dye solution in turn. All the samples' final volumes were increased to 10 mL by adding double-distilled water. To examine the function of individual components, control experiments were conducted concurrently without NaBH_4_ or PdNPs. All the reactions' UV-visible absorption spectra were noted to track any changes in the dyes' spectra.^37^ Values of absorbance are directly correlated with dye concentration.^38^ The kinetics of dye degradation was evaluated with the help of following equation:

1 Degradation (%) = (*A*_0_ − *A*/*A*_0_) × 100

where *A*_0_ is the original absorbance and *A* is the absorbance at the time *t*.

## 3. Results and discussion

### 3.1 UV-vis spectra analysis

**Fig. 4:**
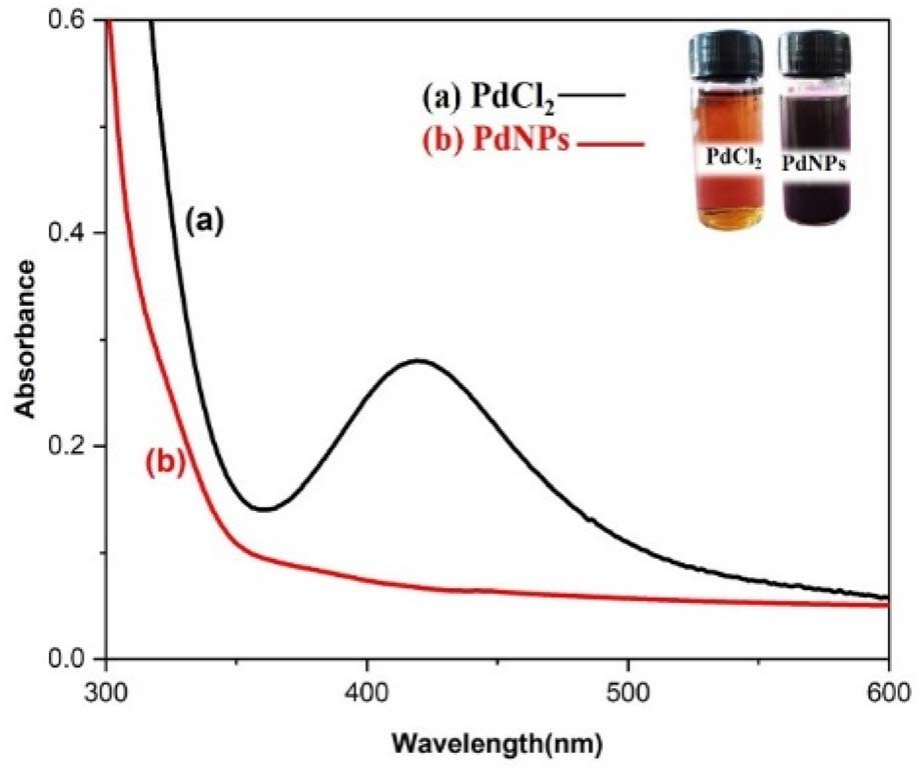
UV-vis absorption spectra of biogenic PdNPs.

Distinctive absorption bands of Pd metal, were investigated both before and after the nanoparticle synthesising process using UV-vis spectroscopy. In the UV-vis wavelength range, the phenomenon called localized surface plasmon resonance (LSPR) gives metal nanoparticles, like Au and Ag, their distinctive colours. On the other hand, palladium nanoparticles' surface plasmon resonance (SPR)^39^ lacks a distinct surface plasmon resonance and instead exhibits a wide, continuous extinction band. The absorption spectra of palladium nanoparticles following three hours of bio reduction by cranberry fruit aqueous extract are shown in Fig. 4, along with the absorption spectra of PdCl_2_ solution for comparison.^40^ The ligand-to-metal charge transfer transition (LMCT) and d–d electronic transitions of the Pd(ii) ions were identified as the cause of the absorption bands seen in the PdCl_2_ spectra at 425 nm, which were caused by the absorption of Pd(ii) ions solution.^41^ The total reduction of Pd(ii) ions was verified by the absence of absorption peaks above 425 nm in the Pd nanoparticle spectrum (Fig. 4). An extensive absorption band that was apparent across the visible range near-ultraviolet region was identified in the PdNPs' spectra.

**Fig. 5:**
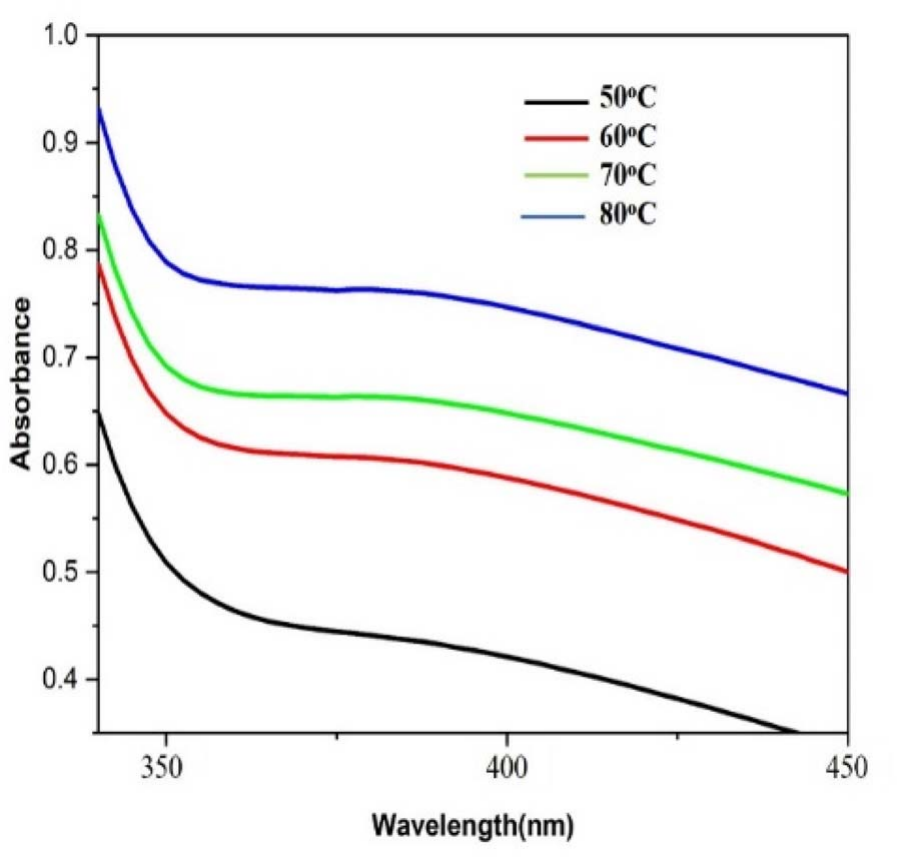
UV-vis absorption spectra for the effect of varying temperature.

**Fig. 6:**
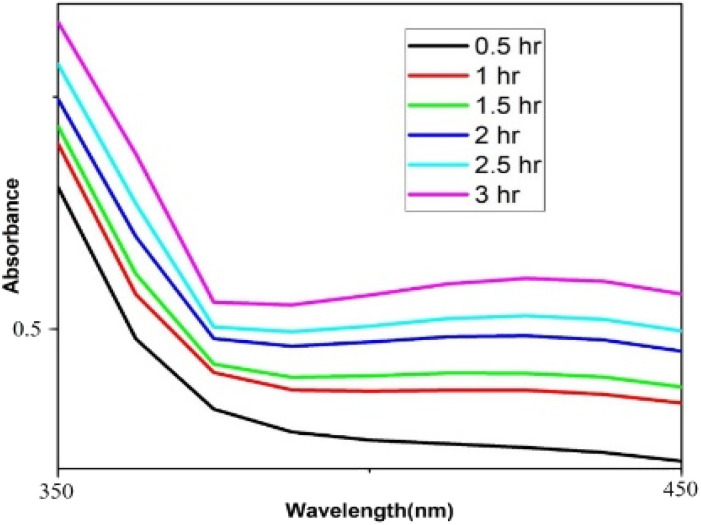
UV-vis absorption spectra for the effect of time of stirring.

The UV spectrum of the colloidal palladium nanoparticles made by varying the reaction temperature is shown in (Fig. 5). The reaction temperatures ranged from 50 °C to 80 °C when 1 mL of aqueous cranberry fruit extract and 1 mM of PdCl_2_ were utilized for the synthesis of the palladium nanoparticles. An effective formation of PdNPs is revealed by the rise in absorbance of the spectra, which is interpreted as an increase in reaction temperature. However, when the temperature is increased further, agglomeration between the palladium particles happens, and the curve's steepness decreases, increasing the size of the palladium particles.Fig. 6 illustrates the impact of reaction time on the synthesis of palladium nanoparticles. The complete reduction of Pd(ii) is shown by the disappearance of a peak at 430 nm, and the rise in absorbance after three hours is likely caused by an increase in the concentration of nanoparticles in the solution. This indicates that the mean particle size of palladium decreases with increasing stirring duration and rate.

**Fig. 7:**
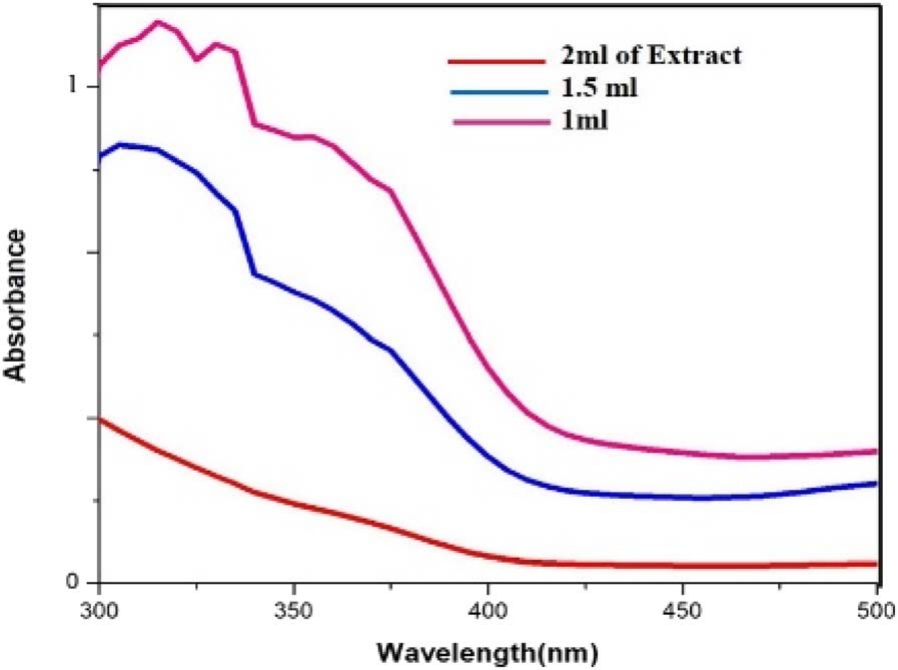
UV-vis absorption spectra for the effect of varying concentration of the extract.

In the process of producing biogenic palladium nanoparticles, the aqueous extract of cranberry fruit serves as a reducing agent. As shown in (Fig. 7), higher extract concentrations result in a steeper curve than lower concentrations, indicating an effective reduction of the palladium nanoparticles. Due to the dilution effect of the extract the rate of the reduction gets lesser at lower concentration.

### 3.2 XRD analysis

**Fig. 8:**
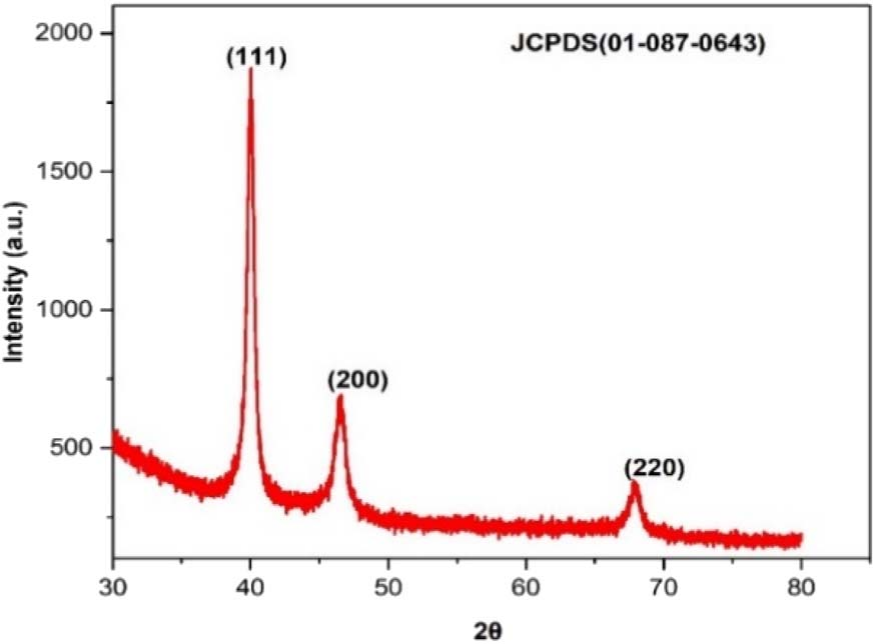
XRD pattern of biosynthesized palladium nanoparticles.

The crystallinity of biogenic PdNPs was investigated using XRD analysis. The diffractogram exhibits three unique reflections at 39.98° (111), 46.49° (200), and 67.95° (220), as seen in Fig. 8. These three reflections show the face-centered cubic (FCC) structure of metallic palladium, which coordinated the standard powder diffraction card of JCPDS palladium file no. 01-087-0643.^40^ Unlike the other two reflections, the strong reflection at (111) may indicate the preferred trajectory for the nanocrystals' development. The Debye–Scherer equation was used to determine the mean crystalline size (∼6.7 nm) of PdNPs based on the half width of the (111) reflection (Table 2).

**Table 2:** XRD parameters of PdNPs synthesized from aqueous cranberry fruit extract

2*θ* of the intense peak (deg.)	*θ* of the intense peak (deg.)	*hkl*	FWHM of intense peak (*β*) radians	*d*-Spacing (nm)	Mean crystalline size (nm)
39.925	19.9625	(111)	385.3122	2.25628	6.711902
46.434	23.217	(200)	0.96885	1.95400	—
67.767	33.8835	(220)	0.66594	1.38169	—

### 3.3 TGA–DT analysis

Thermogravimetric analysis (TGA) was used to comprehend the degradation pattern and thermal consistency of PdNPs. To assess the stability of biogenic PdNPs, thermogravimetric tests were conducted in a nitrogen atmosphere at a heating rate of 10 °C per minute. The differential thermal analysis curve was then produced based on the thermal degradation pattern that was discovered.^41^ The thermal degradation pattern indicates that the palladium metal NPs exhibit stability up to 600 °C and the loss in weight around ∼37.32%. The differential thermal analysis (DTA) curve's initial weight loss of 5.05% might be the result of water molecules evaporating because of surrounding moisture adsorption.

**Fig. 9:**
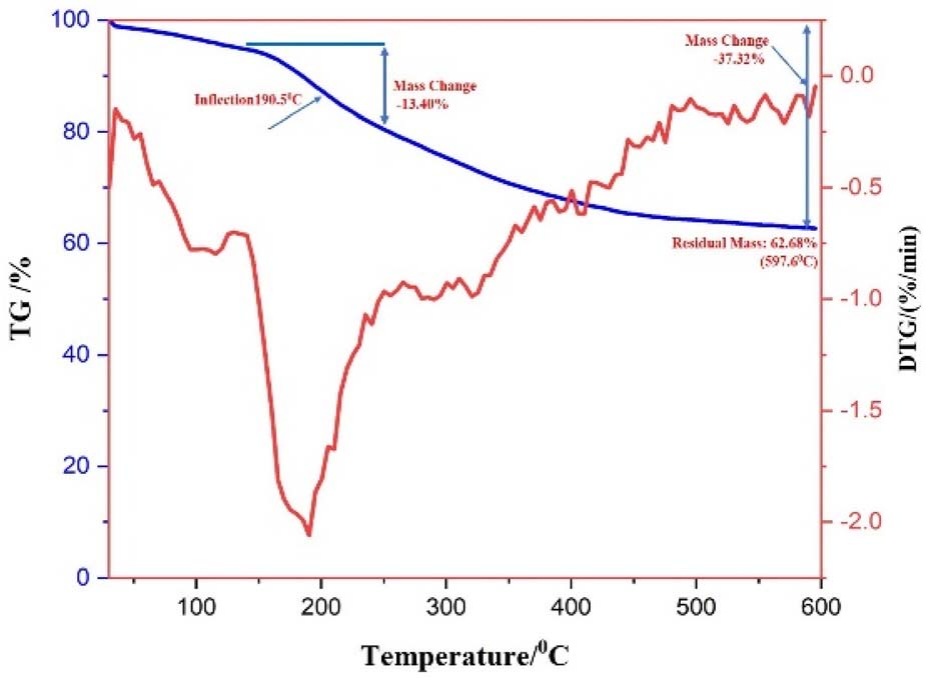
TGA and DTA pattern of biogenic PdNPs.

The extreme decomposition of the PdNPs occurs in the temperature series of 190.5 °C–520 °C, and in this temperature range, the weight reduction is approximately 13.40%, the breakdown of the phytochemicals from the plant extract (PE), which are present on the surface of the NPs and function as stabilizing agents, may be the cause of this reduction in weight. When the as-prepared catalyst was subjected to thermogravimetric investigation up to 600 °C, a total weight loss of 37.32% was recorded, which can be ascertained that the PdNPs are not only reduced in size but also have a good thermal firmness.^42^Fig. 9 displays the DTA curve and the TG pattern.

### 3.4 XPS analysis

**Fig. 10:**
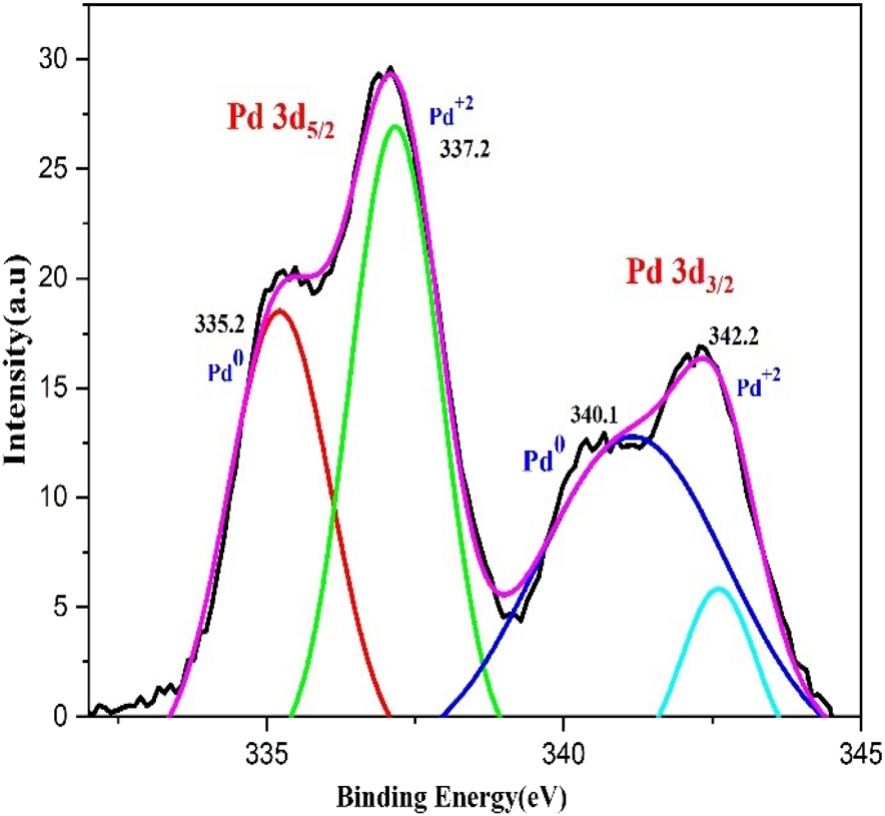
XPS spectra of the Pd 3d region of the biogenic PdNPs.

XPS analysis was used to examine the bio fabricated PdNPs' oxidation status. Two doublets, Pd 3d_3/2_ and Pd 3d_5/2_, can be seen in the 3D deconvoluted spectra of the PdNPs synthesized from aqueous cranberry fruit extract (Fig. 10). This doublet's appearance indicates that Pd exists in two distinct states. Pd 3d_3/2_ (*i.e.*, 340.1 eV and 342.2 eV) and Pd 3d_5/2_ (*i.e.*, 335.2 eV and 337.2 eV) have corresponding binding energies (BE) that indicate that green-synthesised PdNPs depart with oxidation state zero, or metallic Pd (Pd^0^) and +2 (Pd^2+^).^43^ Therefore, it is plausible to assume that the amount of polyphenol components in the aqueous cranberry fruit extract contributed to the reduction of Pd(ii) to elemental palladium.^44^

### 3.5 SEM and TEM analysis

**Fig. 11:**
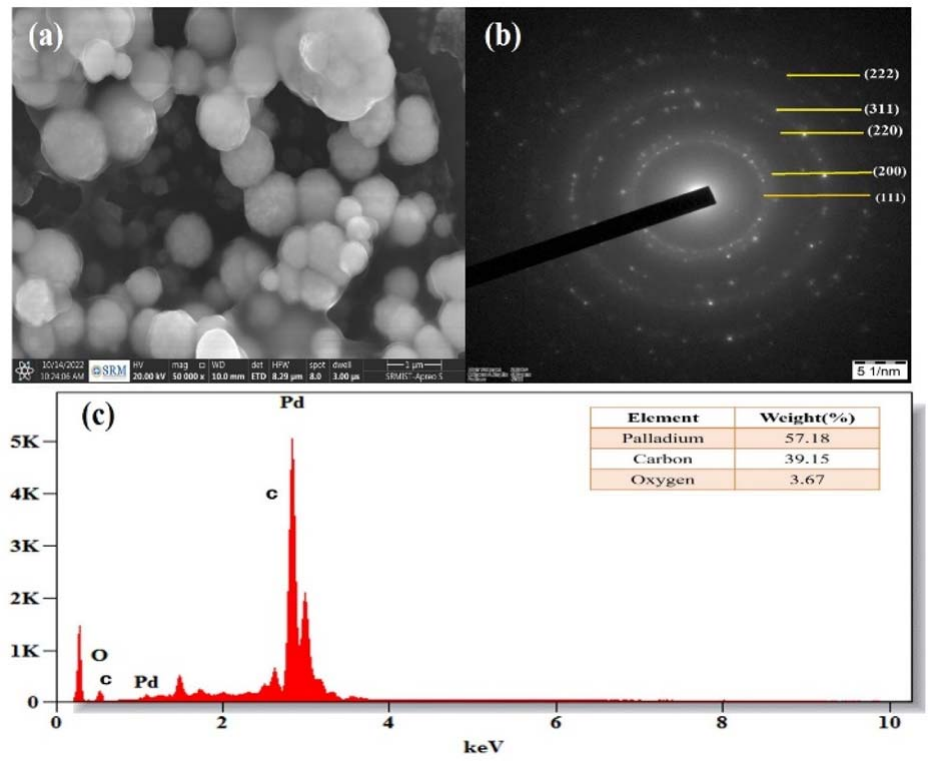
(a) SEM image of PdNPs at 500 nm, (b) selected area electron diffraction (SAED) pattern, (c) energy dispersive X-ray spectrum of biosynthesized palladium nanoparticles.

**Fig. 12:**
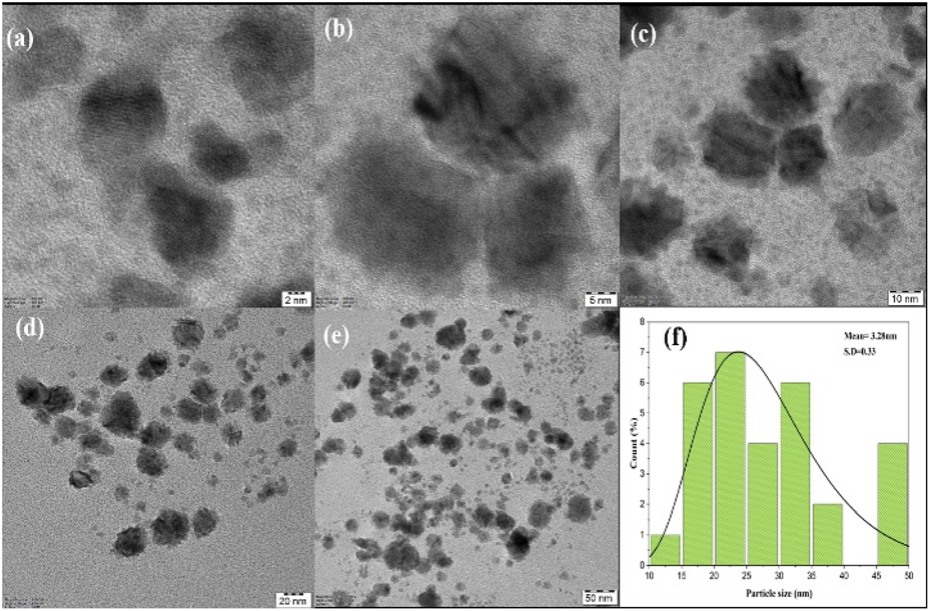
(a–e) HRTEM biosynthesized palladium nanoparticles at 2 nm, 5 nm, 10 nm, 20 nm and 50 nm scale. (f) Histogram of particle size distribution graph (3.28 nm).

Field emission scanning electron microscopy (FE-SEM) was used to examine the morphology of the PdNPs, and Fig. 11a image reveals the PdNPs' spherical shape. The size distribution and structure were investigated with transmission electron microscopy (TEM). On the other hand, smaller nanoparticles with a homogeneous morphology are produced when PdNPs are synthesized under optimal conditions.^45^ As seen by the histogram plot in Fig. 12f, the spherical-shaped nanoparticles really had a mean particle size distribution of 3.2 nm. Structures formed by assembled tiny crystalline spheres that ultimately form a regular,^46^ lengthy, and disaggregated net are revealed by HRTEM images Fig. 12a–e.

Energy dispersive X-ray spectroscopy was used to analyse the elemental composition of PdNPs, Fig. 11c. With a high optical absorption peak at 3.1 and 3.6 keV respectively, the presence of pure metallic PdNPs with 57.18% is confirmed.^47^ The presence of carbon with 39.15% is due to the biomass from aqueous cranberry fruit extract used for the generation of the PdNPs. The selected area electron diffraction (SAED) pattern's brilliant circular spots, as seen in the Fig. 11b, are indexed and indicate that the metal nano particles are single, crystalline in nature.

## 4. Biological activity

### 4.1 Antibacterial activity

**Fig. 13:**
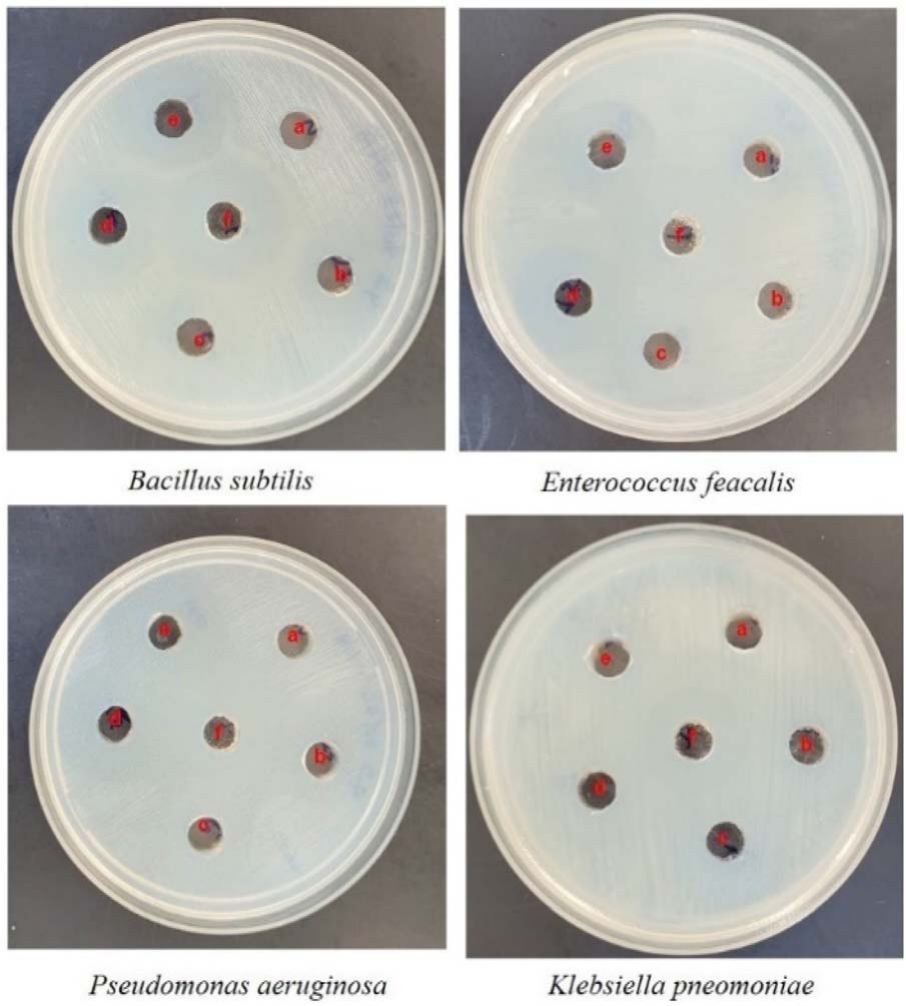
Antibacterial study of biogenic PdNPs against pathogenic Gram-positive (*Bacillus subtilis*, *Enterococcus faecalis*) and Gram-negative (*Pseudomonas aeruginosa*, *Klebsiella pneumonia*). (a) 0 μg per well, (b) 50 μg per well, (c) 100 μg per well, (d) 150 μg per well, (e) 200 μg per well, (f) azithromycin (30 μg mL^−1^).

Well diffusion assay was used to ascertain, the antibacterial activity of the biogenic PdNPs. As test bacterial strains, two Gram-positive (*Bacillus subtilis, Enterococcus faecalis*) and Gram-negative (*Pseudomonas aeruginosa*, *Klebsiella pneumonia*) strains were employed. The development of a clear zone surrounding the PdNP-inoculated well in each plate after a 24 hour incubation period attested to the antibacterial activity (Fig. 13). Aqueous cranberry fruit extract, from which PdNPs were generated, shown strong antibacterial activity against Gram-positive and Gram-negative bacterial strains. The primary mechanism of metal nanoparticles' antibacterial activity has been claimed to be related to internalization and interaction of nanoparticles with cell membranes, which interferes with transport; alternatively, it may be caused by interactions with enzymes, DNA and respiratory system suppression.

**Fig. 14:**
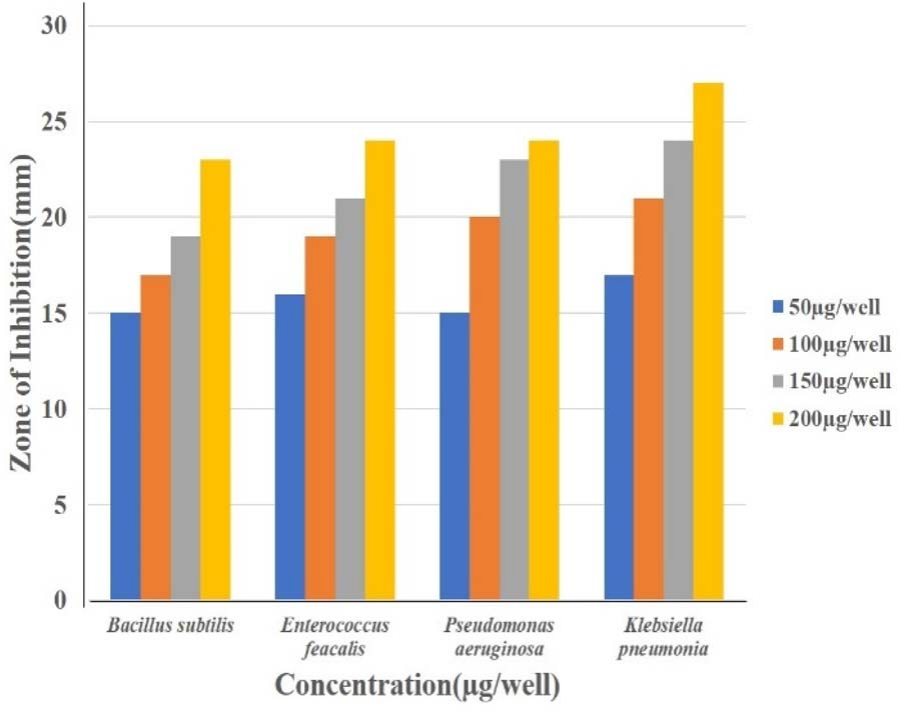
Histogram of antibacterial activity of biosynthesized PdNPs.

The results of this investigation demonstrated that PdNPs have more potential for bactericidal activity against Gram-negative bacteria than against Gram-positive bacteria, (Table 3) which is in line with other findings.^48^ PdNPs generated from aqueous cranberry fruit extract had substantial bactericidal action against Gram negative bacteria in this work (Fig. 13 and 14). This may be because PdNPs may more quickly and easily penetrate the thin outer membrane of Gram-negative bacteria to block their metabolic processes than they can *via* the thicker membrane of Gram-positive bacteria.^49^ Additionally, the bactericidal action against Gram negative bacteria may be influenced by phytochemicals found in the extract that serve as a capping agent on the surface of PdNPs.

**Table 3:** Histogram of antibacterial activity of biosynthesized PdNPs

Name of the organism	Zone of inhibition (mm)					ZOI (mm) standard (azithromycin)
	0 μg per well	50 μg per well	100 μg per well	150 μg per well	200 μg per well	30 μg per well
*Bacillus subtilis*	—	15	18	19	21	25
*Enterococcus feacalis*	—	14	16	19	20	26
*Pseudomonas aeruginosa*	—	14	19	23	25	24
*Klebsiella pneumonia*	—	17	20	22	26	26

### 4.2 Cytotoxic activity of palladium nanoparticles

The cytotoxic effect of the biosynthesized PdNPs at various concentrations was evaluated using the MTT test on MCF-7 breast cancer cell lines. The proliferation of MCF-7 cancer cells is significantly inhibited by biogenic PdNPs, surpassing the effectiveness of the conventional medication doxorubicin.

MCF-7 cells were exposed to biogenic PdNPs at varying doses (0 μg mL^−1^–15 μg mL^−1^) for 48 hours and the mixture was then incubated at 37 °C in a humidified CO_2_ incubator containing 5% CO_2_. Using the MTT assay, the percentage of inhibition of cell growth was ascertained.^50^

**Fig. 15:**
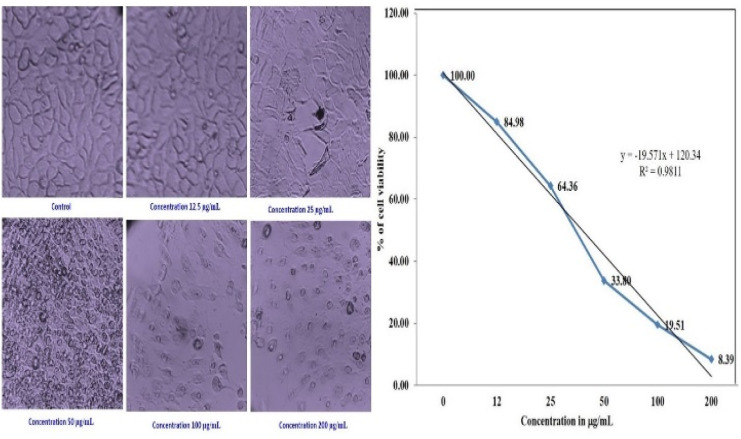
Morphology showing the cytotoxic effect of the PdNPs on the MCF-7 cell line and dose–response curve (DRC).

The cells' viability dramatically decreased as the dosage concentration was increased from 12 to 200 g mL^−1^. To regulate the concentration (IC_50_) that constrains cell growth by 50%, the dose–response curve (DRC) (Fig. 15), was employed using statistical analysis, the IC_50_ value of PdNPs for the MCF-7 cell line was determined to be 68.88 μg mL^−1^. As seen in (Fig. 14), the MCF-7 cells' microscopic pictures were captured at various palladium nanoparticle concentrations.

### 4.3 Investigation of catalytic activities of biogenic PdNPs

#### 4.3.1 Sunset yellow (SY) and indigo carmine (IC) degradation

Azo dyes like sunset yellow (SY) and anionic dyes like indigo carmine (IC) are commonly used as food adulterants and in textile sectors. In SY and IC, the primary chromophores are 6-hydroxy-5-[(4-sulfophenyl) azo]-2-naphthalenesulfonic acid and 5,5′-indigodisulfonic acid, respectively.

**Fig. 16:**
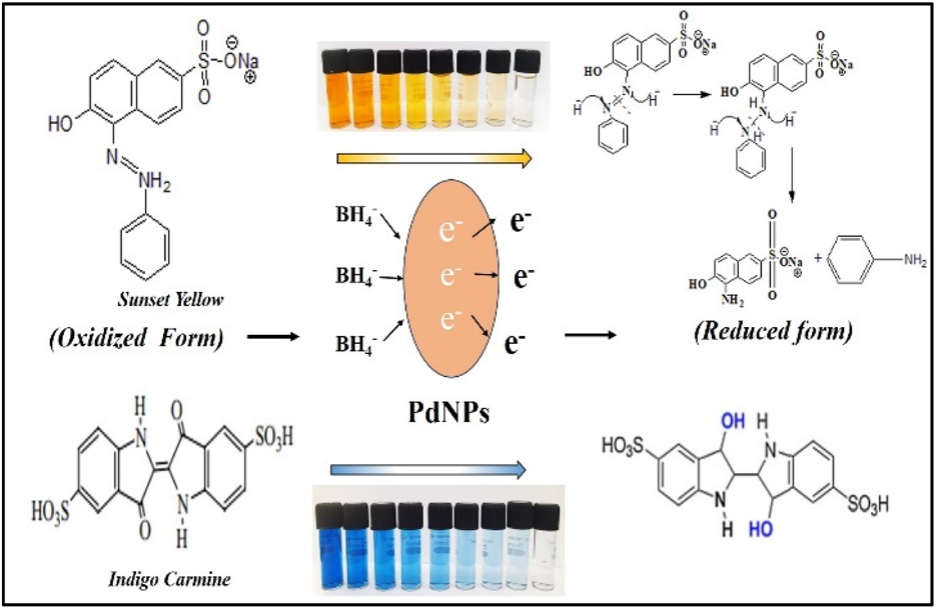
Possible mechanism of degradation of sunset yellow and indigo carmine by NaBH_4_ with PdNPs catalyst.

**Fig. 17:**
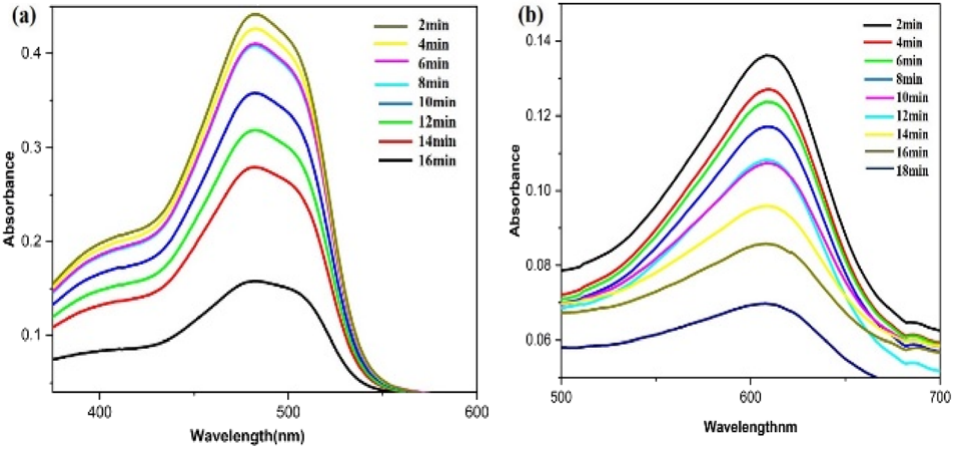
UV-vis spectra for the catalytic reduction of (a) sunset yellow, (b) indigo carmine in the presence of biogenic PdNPs.

Fig. 16 illustrates the possible mechanism for the PdNPs' photocatalytic activity for the degradation of SY and IC in the presence of NaBH_4_. The various reaction conditions for the effective degradation were also carefully examined, including pH, photocatalyst dose, and dye concentration.^51^ For catalytic degradation experiments, the distinctive optical absorption peak for SY at 483 nm and IC at 610 nm was examined (Fig. 17a and b). The goal of optimizing NaBH_4_ and PdNPs concentrations is to maximize degradation with the least amount of catalyst and reducing agent.

It is evident that PdNPs act as electron carriers, enabling hydroxide ions to participate in the cleavage of bond and produce smaller, harmless fragments. By passing off electrons to the dye molecules from NaBH_4_ as donor *via* mediator Pd nano-catalyst, or electrons from NaBH_4_ moving *via* PdNPs as mediator and forming hydride ions which cleaves dyes to non-toxic species, the nano-catalyst aids in the electron-shuttling process when added to the reaction mixture. Eventually, the absorption of dye intensity disappears within the allotted time. Initially, the degradation processes were observed in the absence of Pd nanoparticles using only NaBH_4_ as a reducing agent. Various quantities of NaBH_4_ (10 mM) were employed to break down the dye, but even after three hours, no total degradation could be accomplished. PdNPs were found to speed the reduction process, as evidenced by the quick decolorization and elimination of the distinctive absorption peak of SY/IC solutions. Palladium nanoparticles function as a mechanism for relaying electrons from donor (BH_4_^−^ ion) to acceptor (SY/IC); this favourable kinetics of electron transfer leads to a rapid reduction in dye. The dye's discolouration signifies the chromophore group splitting.

#### 4.3.2 The effect of biogenic PdNPs catalyst's dosage

**Fig. 18:**
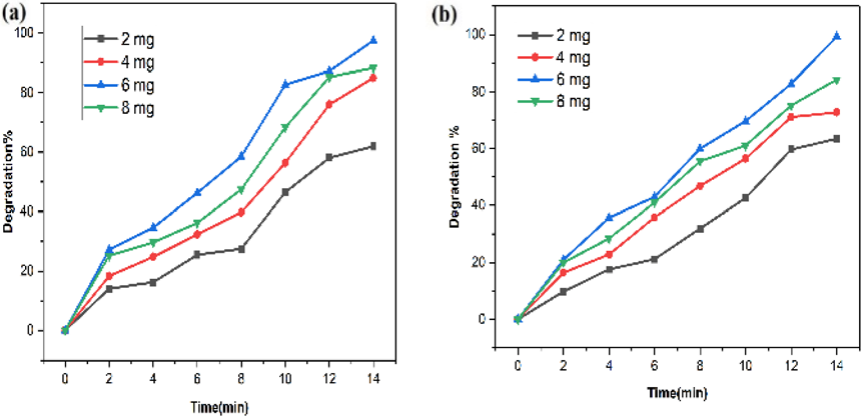
Effect of photocatalyst dose on (a) SY and (b) IC photodegradation using biogenic PdNPs photocatalyst.

The impact of photocatalyst quantity on SY/IC photodegradation was assessed by adjusting the amount of PdNPs from 2 mg to 8 mg in the presence of 2 ppm of dyes SY and IC along with 10 mM of NaBH_4_ under UV light (Fig. 18). This allowed for an examination of the influence of nanoparticle dosage over dye degradation. The photocatalyst dose had a significant impact on the photodecomposition of SY/IC, as the results showed. It is evident that when the photocatalyst dose was increased, the degradation of SY dye increased from 61% to 97%, while for IC, it increased from ∼63% to ∼99%. The addition of more photocatalytic active sites to the medium, which have the capacity to produce more radical ions, is directly responsible for this improvement in the degradation.^52^

However, because there is a greater surface area accessible for catalysis, the dye degradation first increases and reaches a maximum at a dosage of 6 mg of nanoparticles. There will not be enough space for the nanocrystals to distribute themselves in the solution as more photocatalyst is added instead, because of the elements' surface energy, the particles may adhere to one another and aggregate. Dye degradation efficiency begins to decline at 8 mg of nanoparticles. As a result, most of the photocatalytic active sites were obstructed, which reduced the system's ability to degrade materials.^53^

#### 4.3.3 Influence of concentration of SY/IC

**Fig. 19:**
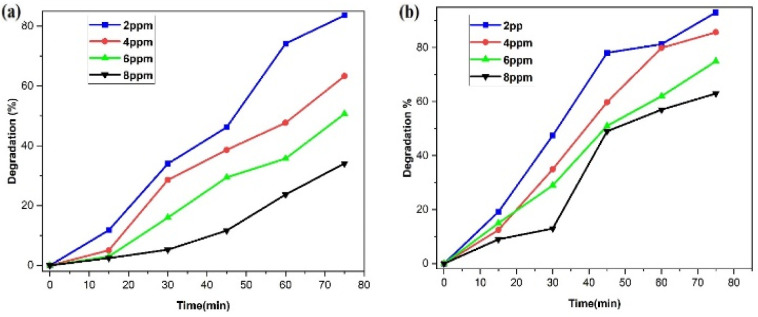
Effect of (a) SY and (b) concentration on the catalytic performance of the PdNPs photocatalyst.

Under UV irradiation, the dye degradation efficiency was investigated regarding the starting dye concentration of SY/IC, ranging from 2 ppm to 8 ppm, while keeping the photocatalyst dose at 6 mg in the presence of NaBH_4_. Fig. 19a and b presents the obtained photocatalytic data graphically. The obtained results showed that at the lowest SY/IC concentration of 2 ppm, the Pd nanoparticle's photocatalytic function was effective. The degradation of IC decreased from 93% to 63% and the degradation of SY gradually decreased from ∼83% to ∼34% when the SY concentration was increased to 8 ppm. This reduction is caused by the photocatalyst surface's reduced ability to absorb light due to a higher dye concentration. This, in sequence, lowers the generation of OH˙ radical ions, that are essential to the photodegradation process.

#### 4.3.4 Influence of pH

**Fig. 20:**
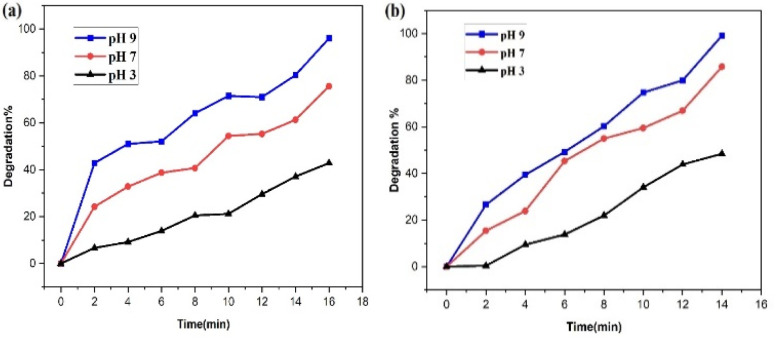
Impact of pH value on the catalytic performance of the PdNPs photocatalyst on the (a) SY and (b) IC photodegradation.

The accessibility of OH˙ radicals in the reaction medium is generally a direct indicator of the photocatalyst's photocatalytic performance, and this enhances the photocatalytic decomposition of SY/IC dyes by several orders of magnitude in alkaline solution. The photodegradation of SY/IC dye in the presence of produced PdNPs photocatalyst at three distinct pH values – 3, 7, and 9 – is depicted in Fig. 20a and b as being dependent on pH value. The outcomes show that increasing the pH value to 9 led to advanced photodegradation,^54^ the lowest degradation performance was obtained at an acidic value pH 3, with approximately 42% degradation of SY and about 48% of IC.

The PdNPs photocatalyst exhibited maximum degrading activity as the solution's pH increased, as was expected. At pH 9, 96% of SY and 99% of IC were broken down. Because SY/IC dyes are anionic, their improved degradation at maximum pH values can be related to the photocatalyst's generation of more OH˙ radical ions, which enhances the efficiency of the photodegradation process.

#### 4.3.5 Kinetic study of photocatalytic degradation of SY/IC

**Fig. 21:**
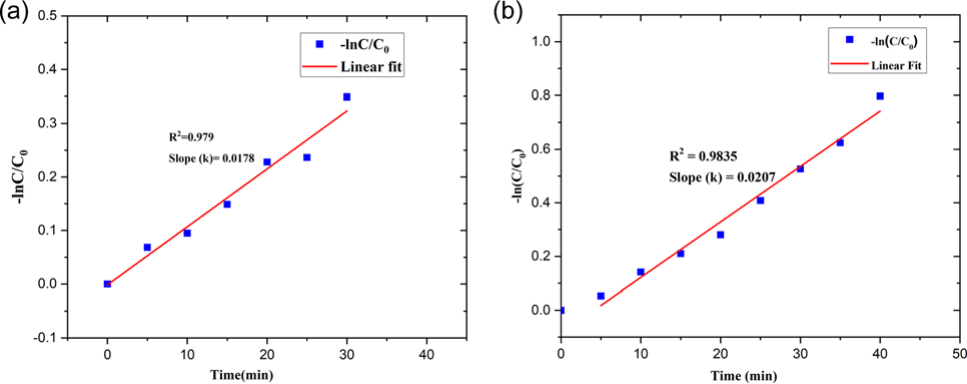
Pseudo-first-order kinetics for (a) SY and (b) IC photodegradation over the PdNPs photocatalyst.

The development of photocatalytic degradation technology for industrial water treatment benefits greatly from the design, scaling up, and optimization of photocatalytic reactors, all of which are made possible by an ideal degradation kinetic expression. The degradation of the substrates through photocatalysis has been examined using Langmuir–Hinshelwood (L–H) rate equations.^55,56^ The pseudo-first-order kinetics of the photocatalytic degradation of SY/IC are demonstrated by the linear relationship of ln *C*_0_/*C vs. t*Fig. 21a and b.

2 ln *C*_0_/*C* = *kt*

where *C*_0_/*C* is the normalized SY/IC concentration, *t* is the reaction time, and *k* is the apparent reaction rate constant (Table 4).

**Table 4:** Photocatalytic degradations of various dyes using biogenic PdNPs from various sources

Source of PdNPs	Dye	Light source	Degradation (%)	Degradation time (min)	Ref.
*Sapium sebiferum* leaf extract	Methylene blue	Visible light	90	70	57
*Andean blackberry* leaf extract	Methylene blue	Sun light	72.16	420	58
*Saccharomyces cerevisiae*	Direct blue 71	UV light	98	60	59
*Pimpinella tirupatiensis* plant extract	Congo red	UV light	94	60	60
*Brown algae*	Congo red	UV light	99.25	10	61
	Methyl orange		99.21	10	
	Methyl red		95.45	10	
*Konjac glucomannan* templated	Methyl orange	UV light	93.4	120	62
	Acid red I		89.1	90	
	Ponceau C		83.2	135	
	Acid violet 7		79.3	150	
	Golden-orange II		77.1	120	
	Acid orange 74		70.1	250	
*Unare gum spinosa*	Congo red	UV light	83	40	63
*Daucus carota* leaves	Rhodamine 6G	UV light	98	30	64
Aqueous cranberry fruit extract	Sunset yellow	UV light	97	16	Present work
	Indigo carmine		99	18	

The kinetics of the photocatalytic degradation reactions are depicted in Fig. 17a and b, and they can be characterized as pseudo first order by ln(*C*/*C*_0_) = *kt*. Plots of ln(*C*/*C*_0_) *vs.* irradiation duration were used to determine the rate constant (*k* × 10^−2^ min^−1^). The intended rate constant for degradation of SY and IC by PdNPs are 1.7 × 10^−2^ min^−1^ and 2.0 × 10^−2^ min^−1^ respectively. The *R*^2^ of pseudo first-order kinetic model for the photocatalytic degradation for PdNPs of SY and IC are 0.979 and 0.9835 respectively.^65^ The catalyst based on the pseudo first-order kinetic model showed a stronger association with photocatalytic degradation of SY and IC, according to the *R*^2^ values.

#### 4.3.6 NMR studies of SY and IC and their photodegraded products

**Fig. 22:**
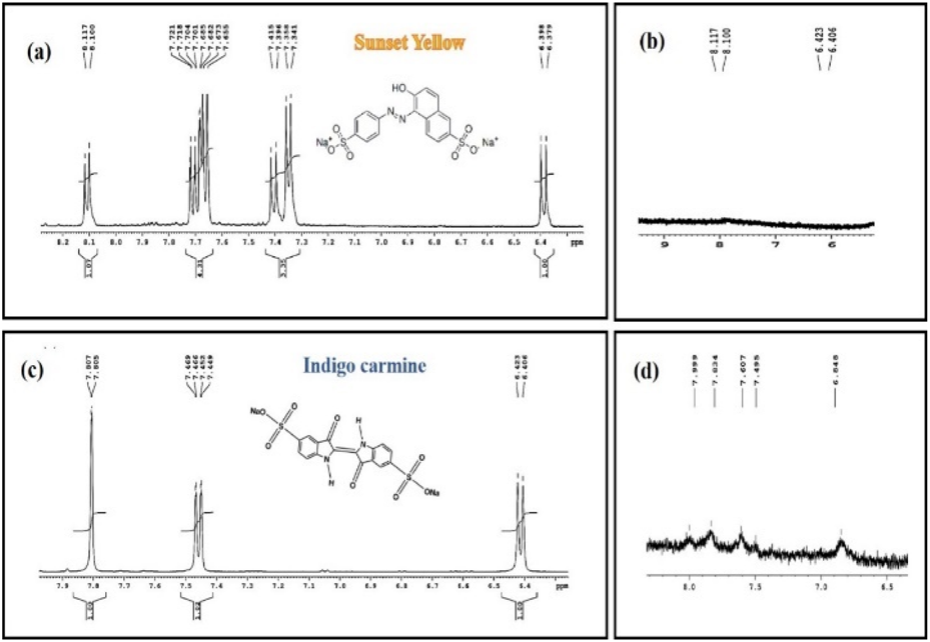
^1^H NMR spectrum of control SY and IC (a and c) and photodegraded SY and IC (b and d).

To support the photodegradation process, a comparative ^1^H NMR analysis of control and the photodegraded product of SY and IC had been conducted (Fig. 22). For this purpose, NMR study was carried out for the control SY and IC with the concentration of 50 mg L^−1^ whereas their degraded products were taken as 0.02 mg L^−1^ along with PdNPs as catalyst, in dosage of 0.5 mg mL^−1^. The time taken for the degradation was 45 min and the ^1^H NMR study was done in the range of 6 to 10 ppm with 125 number of scans. D_2_O served as the NMR solvent.

It is known that the characteristic NMR signals of the control SY and IC dye are found to be present downfield at the range of 6 to 8 ppm (aromatic region) in the ^1^H NMR spectra (Fig. 22a and c). The distinctive aromatic protons of aniline and naphthalene moieties for the SY dye were confirmed by the peaks at 7.72,7.76 and 7.34 ppm respectively, while the characteristic aromatic protons of oxindole moiety for the IC dye showed peaks at around 7.74 ppm, 7.78 ppm, and 7.44 ppm next to the –NH group.^66^

As expected from the hypothesized mechanism (Fig. 16), the degraded products have weak aromatic proton signals with a change in chemical shift and splitting pattern following the reduction process. The reduced SY product shows faint aromatic proton signals, perhaps due to the presence of degraded aniline and sulphonate naphthalene moieties (∼6.64 ppm, ∼6.73 ppm) with chemical shifts at 6.406 ppm and 6.423 ppm. The NMR spectra for the reduced product of IC revealed modest aromatic signals for SO_3_H attached 3-oxyindole moiety protons (∼8.226 ppm, ∼7.79 ppm), with chemical shifts at 7.9 ppm and 7.83 ppm.

Thus, the reduction of SY and IC is confirmed by the proton NMR analysis of the photodegraded products, with the signals of characteristic peaks is either absent or with modified chemical shifts, associated with degraded products still containing aromatic moiety.

## 5. Conclusion

Green techniques, such as combining an aqueous cranberry fruit extract with PdCl_2_ solution, were effectively assisted to create palladium nanoparticles with strong confirmation from UV-visible spectrum analysis with the elimination of the SPR peak. XRD examination verified the crystalline nature and face-centered cubic structure of PdNPs. FEG-SEM with EDS and HR-TEM images of the biosynthesized PdNPs proved that the particles had a spherical form, with a particle size range of 2 to 50 nm, with mean particle size distribution of 3.2 nm. The results also confirms that the PdNPs are in significant crystallinity and stability in nature. Gram-positive (*Bacillus subtilis*, *Enterococcus faecalis*) and Gram-negative (*Pseudomonas aeruginosa*, *Klebsiella pneumonia*) bacteria were tested for the antibacterial activity of PdNPs generated from aqueous cranberry fruit extract, and the results showed a notable zone of inhibition. Compared to standard treatment, biogenic PdNPs showed significant cytotoxic effect against MCF-7 cells, with an IC50 value of 68.88 μg mL^−1^. Biogenic PdNPs also underwent testing as a photocatalyst to evaluate its ability to degrade hazardous food adulterants and industrial effluents, such as sunset yellow and indigo carmine.

Under UV light, the photocatalyst demonstrated remarkable degradation efficiency, degrading SY by 97% in 16 minutes and IC by approximately 99% in 18 minutes. Additionally, it was shown that the photocatalyst works best for SY/IC dye degradation in the concentration range of 2 to 8 ppm and that pH 7 and pH 9 are optimal for the best catalytic activity. The degradation follows a pseudo-first-order reaction kinetics, according to the catalyst's kinetic study and their degradation was also confirmed with NMR studies.

## Data availability

Data for this article, including spectrum, figures, and data are available at Science Data Bank at https://www.scidb.cn/en/s/JrM3Mr.

## Conflicts of interest

There are no conflicts to declare.
